# ITGA2 Mediates the Resistance of Hepatocellular Carcinoma to Lenvatinib by Activating the AKT/FOXO3A Signaling Pathway

**DOI:** 10.3390/cancers17172846

**Published:** 2025-08-29

**Authors:** Kai Gong, Bin Xu, Lian Gong, Ming Zhong, Chun Han, Yuechuan Liu, Zeli Yin, Xiangnan Liang, Qiuxiang Wang, Genhua Ye, Binwen Sun, Liming Wang

**Affiliations:** 1 Engineering Research Center for New Materials and Precision Treatment Technology of Malignant Tumors Therapy, The Second Affiliated Hospital, Dalian Medical University, Dalian 116023, China; gongkai001231@163.com (K.G.);; 2 Engineering Technology Research Center for Translational Medicine, The Second Affiliated Hospital, Dalian Medical University, Dalian 116023, China; 3 Division of Hepatobiliary and Pancreatic Surgery, Department of General Surgery, The Second Affiliated Hospital of Dalian Medical University, 467 Zhongshan Road, Dalian 116023, China; 4 Department of Radiotherapy, The Seventh Afffliated Hospital of Sun Yat-Sen University, Shenzhen 518107, China; 5 Department of General Surgery, The Seventh Afffliated Hospital of Sun Yat-Sen University, Shenzhen 518107, China

**Keywords:** hepatocellular carcinoma, lenvatinib, drug resistance, integrin, E7820

## Abstract

**Background/Objectives:** Lenvatinib resistance is an important contributor to poor prognosis in patients with hepatocellular carcinoma (HCC). The correlation between integrin subunit and lenvatinib resistance needs further exploration. This study investigated the role and molecular mechanism of integrin subunit ITGA2 in lenvatinib resistance. **Methods:** Bioinformatics analyses (GSCA, CTRP, GEPIA) revealed that ITGA2 expression was significantly correlated with lenvatinib resistance and that patients with high ITGA2 expression had a poor prognosis. Cell Counting Kit-8 (CCK-8) and Western blotting (WB) experiments confirmed the correlation between ITGA2 expression and the IC50 of lenvatinib in hepatoma cell lines. Cell experiments, including WB, CCK-8 assay, colony formation assay, and flow cytometry, suggested that ITGA2 mediates drug resistance by promoting cell proliferation and inhibiting apoptosis. **Results:** ITGA2 expression knockdown enhanced lenvatinib-induced apoptosis, G1 phase arrest, and proliferation inhibition, whereas its overexpression had the opposite effect. In vivo experiments indicated that ITGA2 overexpression promotes tumor growth in transplantation, whereas its knockdown inhibits tumor progression. The ITGA2 inhibitor E7820 can promote lenvatinib-induced apoptosis and cell cycle arrest in hepatoma cells, thereby reversing lenvatinib resistance. Rescue experiments suggested that *ITGA2* KO or using E7820 to inhibit AKT/FOXO3A phosphorylation can increase the efficacy of lenvatinib, and the pathway activator 740Y-P can reverse the phenotype. ITGA2 overexpression promotes AKT/FOXO3A phosphorylation and reduces the efficacy of lenvatinib, whereas an inhibitor (LY294002) can reverse this phenotype. **Conclusions:** In summary, ITGA2 is a key driver of lenvatinib resistance in HCC and activates the AKT/FOXO3A signaling pathway to promote the resistance of hepatomas to lenvatinib. Targeting ITGA2 can significantly increase the efficacy of lenvatinib and provides a new strategy to overcome liver cancer resistance.

## Simple Summary

Patients with hepatocellular carcinoma often develop drug resistance to lenvatinib. This study explores the role of integrin subunit ITGA2 in this resistance, aiming to confirm how ITGA2 contributes to resistance and its underlying molecular mechanisms. Results indicate that high ITGA2 levels increase lenvatinib resistance in liver cancer cells. Reducing ITGA2 enhances drug efficacy, promoting apoptosis and inhibiting cell growth, whereas increasing ITGA2 reduces efficacy. Mechanistically, ITGA2 induces resistance by activating the AKT/FOXO3A pathway. The inhibitor E7820 can block ITGA2, thereby reversing resistance. This study provides a novel therapeutic strategy of targeting ITGA2 to enhance lenvatinib efficacy, potentially contributing to more effective liver cancer treatments.

## 1. Introduction

The global incidence of primary liver cancer ranks sixth among malignant tumors, while its mortality rate ranks third and is the second leading cause of cancer-related deaths in China [1,2]. The high incidence and mortality rates of liver cancer seriously threaten human health and increase social and economic burdens. Primary liver cancer is classified into three principal pathological subtypes: hepatocellular carcinoma (HCC), intrahepatic cholangiocarcinoma (ICC), and combined hepatocellular-cholangiocarcinoma (cHCC-CCA). Among these, HCC is the most prevalent, accounting for approximately 75% to 85% of cases [3]. Consequently, the present study focuses specifically on HCC. Clinical management of HCC involves a multimodal comprehensive approach, which encompasses surgical resection, liver transplantation, local ablation, transarterial chemoembolization (TACE), molecular targeted agents, and immunotherapy. Nonetheless, due to the insidious nature of early-stage liver cancer symptoms, fewer than 30% of patients are eligible for curative therapies at the time of initial diagnosis [4]. Systemic antitumor treatment plays an important role in the treatment of advanced liver cancer. Molecular targeted therapy is indispensable in systemic antitumor treatment. Multiple target tyrosine kinase inhibitors (TKIs), such as sorafenib and lenvatinib, are the first-line standard treatment drugs for advanced HCC [5,6]. However, one of the main challenges in the treatment of HCC is TKI resistance [7], which directly leads to a low objective response rate, short progression-free survival, and ultimately treatment failure and disease progression. Therefore, in-depth exploration of the molecular mechanism of TKI resistance in HCC patients is crucial for overcoming resistance, developing new sensitization strategies, and improving the prognosis of patients with advanced HCC.

Integrins, a large class of transmembrane receptors that mediate cell–cell and cell–extracellular matrix recognition and adhesion, play a central role in integrating extracellular stimuli with intracellular signal transduction [8]. In addition to mediating adhesion, integrins are widely involved in regulating the proliferation, survival, migration, and metastasis of cancer cells [9]. Dysfunction of these genes is widely speculated to be associated with resistance to various cancer treatments, including TKIs [10]. Integrins are heterodimers composed of α and β subunits; at least 18 α subunits and 8 β subunits have been identified in humans, which can combine to form 24 different integrin receptors [11]. The diverse range of integrin subunits and their multifunctional nature indicate that numerous members warrant deeper exploration regarding their roles in cancer initiation, progression, and modulation of treatment responses. For example, studies have demonstrated that in non-small cell lung cancer, ITGA5 promotes resistance to icotinib by inducing phosphorylation of the STAT3/AKT signaling pathway [12]. In patients with pancreatic adenocarcinoma, ITGA2, a key regulator of the extracellular matrix (ECM), is involved in regulating gemcitabine resistance [13]. In the field of hepatocellular carcinoma, several studies have suggested that integrins are closely related to TKI resistance; for example, ITGB8 mediates AKT stabilization through HSP90 and enhances the regulation of lenvatinib resistance through the AKT signaling pathway [14], and the overexpression of ITGA6 can increase the resistance of acute lymphoblastoid B cells to erlotinib [15]. In addition, the expression levels of ITGA5 and ITGB1 may serve as potential biomarkers for predicting the prognosis of HCC patients receiving sorafenib [16]. Although many studies have investigated integrins and integrin subunits related to malignant tumors, considering that humans have at least 26 integrin subunits, the roles of most of these subunits in HCC patient TKI resistance have not yet been clarified, especially the specific subunits related to resistance to specific TKIs (such as lenvatinib); therefore, their mechanisms must be further explored.

Against the above background, the role of integrin subunits in TKI resistance in HCC patients remains unclear. This study aimed to systematically screen the integrin subunits that are significantly associated with TKI resistance and poor prognosis in HCC patients. To this end, we conducted a systematic bioinformatics analysis to explore public databases and discovered that ITGA2 is significantly associated with poor prognosis and TKI resistance in HCC patients. Through in vitro and in vivo functional experiments, we confirmed that ITGA2 significantly promotes lenvatinib resistance in HCC cells. At the mechanistic level, we found that ITGA2 expression activates the AKT signaling pathway, promoting the phosphorylation of downstream FOXO family effectors and thereby mediating lenvatinib resistance. More importantly, we applied an ITGA2-specific small molecule inhibitor in combination with lenvatinib, which effectively reversed the lenvatinib resistance phenotype in HCC patients. These findings not only confirm that ITGA2 may be one of the key drivers of lenvatinib resistance in HCC patients but also that the synergistic effect of ITGA2-specific small molecule inhibitors combined with lenvatinib provides new therapeutic strategies and a theoretical basis for future clinical applications and resistance reversal.

## 2. Materials and Methods

### 2.1. Bioinformatics Analysis

Using the GSCA tool and drug sensitivity data from the CTRP database, we assessed the drug-resistance correlations between various drugs and integrin subunits, and generated a correlation heatmap using R. (https://guolab.wchscu.cn/GSCA/#/, accessed on 1 September 2024). The public database Gepia2 (http://gepia2.cancer-pku.cn/#/index, accessed on 2 September 2024) was used to identify correlations between genes and prognosis.

### 2.2. Cell Culture and Transfection

The cell lines used in this study, including Hep3B, Huh7, MHCC97H, HepG2, and SNU449, were obtained from the Cell Bank of the Chinese Academy of Sciences (Shanghai, China), with each line authenticated by STR profiling. SNU449 cells were cultured in RPMI 1640 medium containing 10% fetal bovine serum (FBS; Gibco, Waltham, MA, USA). Both Hep3B and HepG2 cells were maintained in MEM supplemented with 10% FBS, whereas MHCC97H and Huh7 cells were grown in high-glucose DMEM with 10% FBS. The lenvatinib-resistant variants, Hep3B-LR and Huh7-LR, were cultured in MEM and high-glucose DMEM, respectively, each medium supplemented with 10% FBS and 10 μM lenvatinib (MCE, Monmouth Junction, NJ, USA). All the cell lines were cultured at 37 °C in a 5% carbon dioxide atmosphere. E7820 is an integrin α2 subunit inhibitor, LY294002 is a PI3K inhibitor, and 740Y-P is a PI3K agonist. All small molecule compounds were purchased from MCE. *ITGA2*-targeted siRNAs (Si162, Si877, Si927, Si1045) and negative control siRNAs were purchased from the GenePharma Company (Suzhou, China). *ITGA2*-targeted shRNAs and negative controls were purchased from the GenePharma Company (Suzhou, China). *ITGA2* overexpression plasmids and negative controls were purchased from GenePharma Company (Suzhou, China). The *ITGA2*-overexpressing lentivirus along with its corresponding negative control were acquired from GeneChem (Shanghai, China). Transfection of HCC cells was performed in accordance with the supplier’s instructions. Subsequently, the transfected cells underwent a two-week selection process using puromycin (2 µg/mL). Overexpression or knockdown efficiency was confirmed through Western blot analysis. Detailed sequence information is provided in Supplementary Methods S1.

### 2.3. Protein Extraction and WB

We extracted total protein from cells and tissues using RIPA lysis buffer (Keygen, Nanjing, China), protein inhibitor solution (Beyotime, Shanghai, China), and phosphatase inhibitor solution (Absin, Shanghai, China). The protein concentration of the lysates was determined using a BCA assay kit (Keygen, Nanjing, China). The denatured proteins in the supernatant of the lysates (20 µg of protein per lane) were separated on an SDS–polyacrylamide gel and then transferred onto a polyvinylidene fluoride (PVDF) membrane (Millipore, Billerica, MA, USA). The PVDF membrane was blocked at room temperature for 15 min with rapid blocking solution (NCM, Suzhou, China) and then incubated with primary antibodies at 4 °C overnight. The PVDF membrane was subsequently incubated with secondary antibody at 37 °C for 1 h. The protein bands were developed using a chemiluminescence imaging system (Servicebio, Wuhan, China) with high-sensitivity chemiluminescent reagent. The protein levels were quantified with image software (Servicebio, Wuhan, China). The detailed information of the antibodies used is shown in Supplementary Methods S2.

### 2.4. CCK-8 Assay

Cell viability was determined using a CCK-8 kit (MCE, Monmouth Junction, NJ, USA). The cells were seeded at a density of 2 × 10^3^ per well in a 96-well plate containing 100 µL of complete medium. The cells were treated and cultured according to the experimental design. After treatment, 10 µL of CCK-8 reagent was added to each well, and the samples were incubated for 2 h. The absorbance was measured at 450 nm using an enzyme reader (Molecular Devices, San Jose, CA, USA).

### 2.5. Colony Formation Assay

The cells were uniformly spread in 6-well plates (2 × 10^3^ cells per well), and lenvatinib was added to the wells according to the experimental design. The cells were cultured at 37 °C in 5% CO_2_ for 2 weeks. When visible colonies were observed, the cells were fixed with 4% paraformaldehyde for 20 min, stained with 1% crystal violet for 20 min, photographed, and counted.

### 2.6. Flow Cytometry for Detecting Apoptosis and the Cell Cycle

After the cells were treated according to the experimental design, apoptosis and the cell cycle were evaluated using flow cytometry. Apoptosis was assessed using an Annexin V-APC/PI apoptosis detection kit (Elabscience, Wuhan, China). After dual staining with Annexin V-APC/PI, the fluorescence levels were measured using a flow cytometer (Agilent, Santa Clara, CA, USA). The apoptosis rate = early apoptosis rate + late apoptosis rate. The cell cycle was evaluated using a cycle detection kit (Keygen, Nanjing, China). After PI staining, the fluorescence levels were measured with a flow cytometer (Agilent, USA).

### 2.7. Animal Experiments

Five-week-old female BALB/c-nude mice were housed in a specific pathogen-free (SPF)-grade animal facility. A total of 5 × 10^6^ cells were subcutaneously inoculated into the mice. After the mice developed palpable tumors, lenvatinib (10 mg/kg) was administered by intraperitoneal injection once a week for two consecutive weeks. The tumor size was measured every 5 days for a total of 5 measurements. All animals were euthanized on the 27th day after inoculation, and tumor tissues were collected. The tumor tissues were photographed and subjected to immunohistochemical detection. The formula for calculating tumor volume was as follows: V (mm^3^) = 1/2 × length × width^2^. The animal experiments were conducted under the guidance of the Institutional Animal Care and Use Committee (IACUC) and received ethical approval from the institutional review board.

### 2.8. Immunohistochemistry and Scoring

The stained sections were evaluated in a blinded manner by two specialized pathologists. A final score was assigned based on the proportion of cells that were positive and the intensity of the staining. The proportion of positively stained cells (negative, 0 points; 1–25%, 1 point; 25–50%, 2 points; 50–75%, 3 points; and 75–100%, 4 points) and staining intensity (unstained, 0 points; weak staining, 1 point; moderate staining, 2 points; strong staining, 3 points) were used to score the positive staining. Finally, the staining index (SI) was calculated according to the following formula: SI = staining intensity score × proportion of positive staining score.

### 2.9. Transcriptome Sequencing

The Huh7-LR cells were divided into the SiNC group and the Si162 group. Total RNA was extracted from the samples, and the mRNA was enriched. A cDNA library was subsequently constructed, and high-throughput sequencing was performed. After comparison with the reference genome and quantification of gene expression, information on gene expression differences and other aspects was analyzed.

### 2.10. Statistical Analysis

All the experiments were conducted with three independent biological replicates. Statistical analysis was performed with GraphPad Prism 9.5.1 (San Diego, CA, USA) software. The specific methods used were as follows. Correlation analysis: A scatter plot was used to show the relationship between the protein expression level of ITGA2 and the corresponding IC50 value of the cell lines, and linear regression analysis was used to evaluate the correlation between the two. Dose–effect relationship: A nonlinear regression model (log(inhibitor) vs. response—variable slope (four parameters)) was used to fit the cell viability data at different drug concentrations. Group comparison: Experimental data (such as Western blot data, colony formation data, etc.) were statistically analyzed for differences between groups on the basis of data characteristics and experimental design via sample *t* tests, one-way ANOVA, or two-way ANOVA (* *p* < 0.05, ** *p* < 0.01, *** *p* < 0.001, **** *p* < 0.0001).

### 2.11. Visualization

The graphical abstract was designed using FigDraw (www.figdraw.com, accessed on 10 July 2025).

## 3. Results

### 3.1. The Expression Level of ITGA2 Is Negatively Correlated with the Prognosis of Liver Cancer Patients and the Sensitivity to Lenvatinib

Integrins are heterodimers composed of 18 α and 8 β subunits that widely regulate tumor cell proliferation, migration, invasion, and response to antitumor drugs [17,18]. However, no comprehensive studies have explored the correlation between integrin subunits and the sensitivity of hepatocellular carcinoma to TKI drugs. Therefore, we used the GSCA platform in combination with the CTRP database to select two first-line TKI drugs (lenvatinib, sorafenib) and two second-line TKI drugs (regorafenib, cabozantinib) to analyze the correlation between drug resistance and each integrin subunit. The screening of integrin subunits was primarily based on their significance level (*p*-value) and effect size (correlation coefficient) in relation to drug resistance, thereby identifying subunits significantly associated with drug resistance. The three subunits with the strongest associations to drug resistance among all integrin subunits (ITGA2, ITGA3, and ITGA6) were selected (Supplementary Figure S1A). Among these, lenvatinib resistance showed the most significant correlation with ITGA2 and ITGA6, whereas sorafenib resistance exhibited the most significant correlation with ITGA3. Further survival analysis (GEPIA) indicated that among these three integrin subunits, the increase in ITGA2 expression was significantly associated with a significant decrease in the overall survival (OS) and progression-free survival (PFS) of patients (highest risk ratio, *p* < 0.05), whereas the associations of ITGA3 and ITGA6 with OS and PFS did not reach the threshold of statistical significance (*p* > 0.05) (Supplementary Figure S1B–D). Therefore, these results suggest that patients with high expression of ITGA2 have a poor prognosis and are significantly associated with lenvatinib resistance.

**Figure 1:**
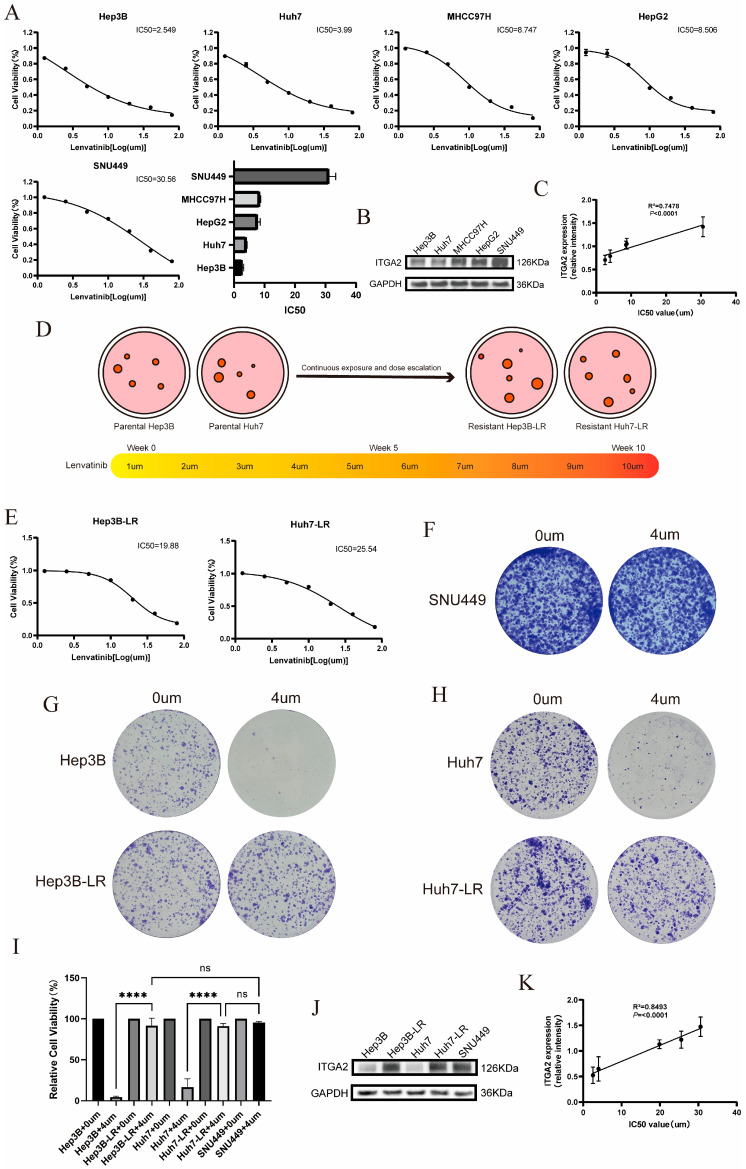
The expression level of ITGA2 is positively correlated with the IC50 value of lenvatinib. (**A**) A CCK8 assay was used to determine the IC50 of lenvatinib in five liver cancer cell lines. (**B**) WB was used to detect the expression level of ITGA2 in five cell lines. (**C**) Linear regression analysis was performed using a scatter plot to correlate the IC50 with the expression level of ITGA2. (**D**) Schematic diagram of the lenvatinib-resistant cell line construction. (**E**) A CCK8 assay was used to determine the IC50 of lenvatinib in Hep3B-LR and Huh7-LR cells. (**F**–**I**) Colony formation experiments were used to detect the colony formation ability of SNU449 (**E**), Hep3B (**F**), Hep3B-LR (**F**), Huh7 (**G**), and Huh7-LR (**G**) cells at concentrations of 0 μM and 4 μM lenvatinib, and statistical analysis (**I**) was conducted. (**J**) WB was used to detect the expression level of ITGA2 in SNU449, Hep3B, Hep3B-LR, Huh7, and Huh7-LR cells. (**K**) A scatter plot of the IC50 values and ITGA2 expression levels was generated, and linear regression was performed. **** *p* < 0.0001. ns, not significant.

To further explore the correlation between the expression level of ITGA2 and the sensitivity of liver cancer to lenvatinib, we conducted a series of cell experiments. First, we determined the IC50 values of lenvatinib for various commonly used hepatocellular carcinoma cell lines using the CCK-8 method (Figure 1A), and the results showed that Hep3B and Huh7 cells were the most sensitive to lenvatinib (with the lowest IC50), whereas SNU449 cells had the strongest resistance (with the highest IC50). Then, we detected the protein expression levels of ITGA2 in these cell lines using Western blotting (Figure 1B), and the results revealed that ITGA2 was expressed at relatively low levels in Hep3B and Huh7 cells, whereas it was relatively highly expressed in SNU449 cells. Regression analysis using scatter plots showed that the lenvatinib IC50 values of liver cancer cells were significantly positively correlated with the expression level of ITGA2 (Figure 1C). To facilitate subsequent experiments for verification, we gradually increased the lenvatinib concentration used to treat Hep3B and Huh7 cells (from 1 μM to 10 μM) and established the corresponding drug-resistant cell lines Hep3B-LR and Huh7-LR (as shown in Figure 1D). The results of the CCK-8 assay confirmed that the IC50 values of the drug-resistant cell lines were significantly higher than those of the parental cells (Figure 1E). The colony formation experiments further confirmed that at 4 μM lenvatinib, Hep3B-LR, Huh7-LR, and SNU449 cells exhibited significant lenvatinib resistance, while the survival rates of Hep3B and Huh7 cells were extremely low (Figure 1F–I). Compared with the expression levels of ITGA2 in Hep3B and Huh7 cells, the expression level of ITGA2 was significantly upregulated in lenvatinib-resistant Hep3B-LR, Huh7-LR, and SNU449 cells (Figure 1J). Linear regression analysis confirmed that the IC50 values of each cell line were significantly positively correlated with the protein expression level of ITGA2 (Figure 1K). Therefore, our results suggest that high expression of ITGA2 is significantly positively correlated with lenvatinib drug resistance.

### 3.2. ITGA2 Promotes the Resistance of Hepatocellular Carcinoma to Lenvatinib

**Figure 2:**
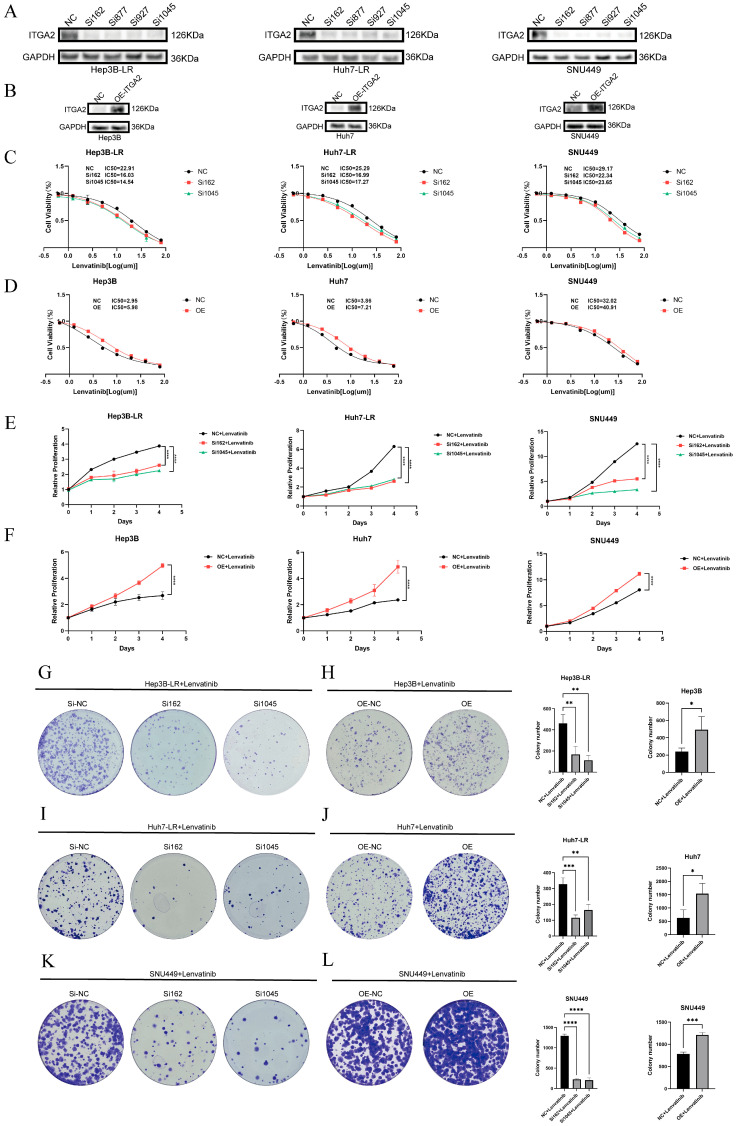
Downregulation of ITGA2 enhances lenvatinib sensitivity and suppresses proliferation in liver cancer cells, while ITGA2 overexpression promotes drug resistance and reduces its inhibitory effects. (**A**) WB analysis of ITGA2 knockdown efficiency in Hep3B-LR, Huh7-LR, and SNU449 cells. (**B**) WB detection of ITGA2 overexpression in Hep3B, Huh7, and SNU449 cells. (**C**) CCK8 assay showing decreased IC50 of lenvatinib after ITGA2 knockdown in resistant cell lines. (**D**) CCK8 assay showing increased IC50 of lenvatinib following ITGA2 overexpression. (**E**,**F**) Cell growth curves from CCK8 assays under ITGA2 knockdown (**E**) or overexpression (**F**). (**G**–**L**) Colony formation assays showing reduced proliferation after ITGA2 knockdown (**G**,**I**,**K**) and increased proliferation after ITGA2 overexpression (**H**,**J**,**L**) in respective cell lines. (**E**–**L**) All the cells were treated with an appropriate concentration of lenvatinib (knockdown groups: Hep3B-LR, Huh7-LR, and SNU449 with lenvatinib at a concentration of 10 μM; overexpression groups: Hep3B and Huh7 with lenvatinib at a concentration of 1 μM; SNU449 with lenvatinib at a concentration of 15 μM). * *p* < 0.05, ** *p* < 0.01, *** *p* < 0.001, **** *p* < 0.0001.

To demonstrate the causal relationship between ITGA2 expression and sensitivity to lenvatinib, we conducted gain-of-function and loss-of-function experiments. ITGA2 was transiently knocked down in resistant cells (Hep3B-LR, Huh7-LR, and SNU449) (Figure 2A), and ITGA2 was overexpressed in sensitive cells (Hep3B, Huh7) and SNU449 cells (Figure 2B), and the transfection efficiency was verified by Western blotting. The results of the CCK8 assay revealed that after ITGA2 was knocked down in resistant cells, the IC50 value of lenvatinib significantly decreased (Figure 2C). Conversely, after ITGA2 was overexpressed in sensitive cells and SNU449 cells, the IC50 value of lenvatinib significantly increased (Figure 2D). These findings confirmed that the expression level of ITGA2 was negatively correlated with the sensitivity of cells to lenvatinib. We further studied the effect of ITGA2 on the ability of lenvatinib to inhibit cell proliferation. The results of the CCK8 assay suggested that, compared with that of the control group, the viability of resistant cells (Hep3B-LR, Huh7-LR, and SNU449) treated with lenvatinib after ITGA2 knockdown significantly decreased (Figure 2E), and the colony formation ability was significantly inhibited (Figure 2G,I,K). In contrast, after ITGA2 was overexpressed in sensitive cells (Hep3B, Huh7) and SNU449 cells, the cell viability (Figure 2F) and colony formation ability (Figure 2H,J,L) under lenvatinib treatment were significantly increased. These results indicate that ITGA2 participates in mediating lenvatinib resistance by promoting cell proliferation.

**Figure 3:**
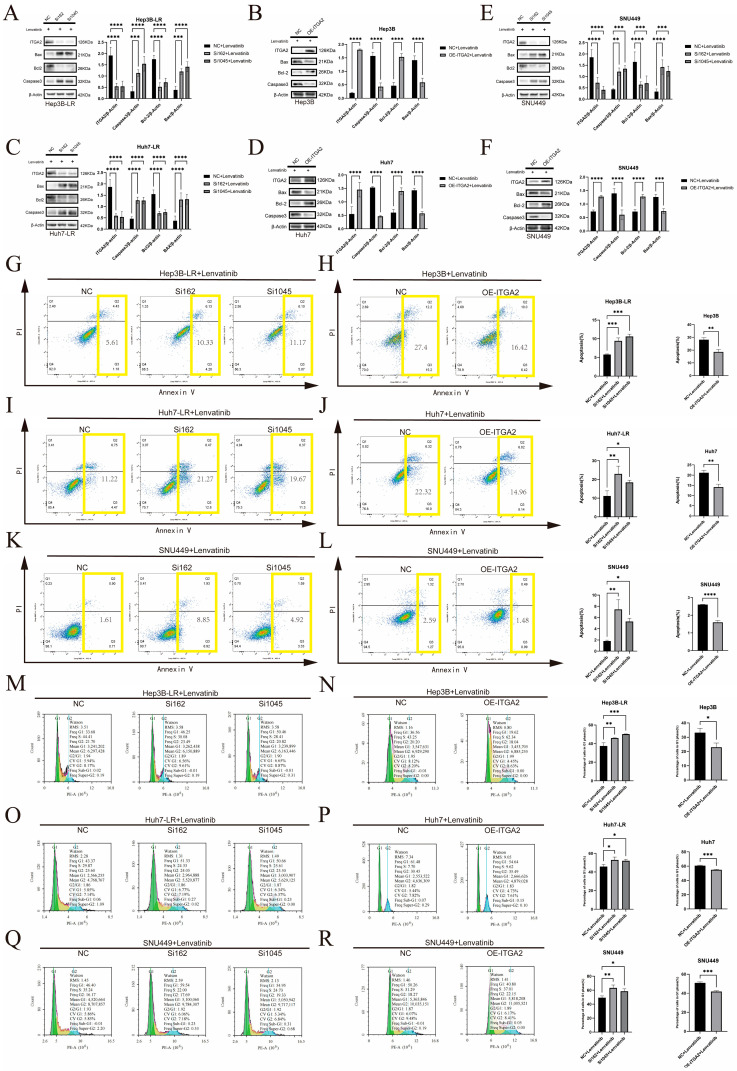
Knocking down ITGA2 can promote the apoptosis and cell cycle arrest of liver cancer cells caused by lenvatinib. (**A**–**F**) WB analysis of ITGA2, Bax, Bcl-2, and Caspase-3 expression after ITGA2 knockdown (**A**,**C**,**E**) or overexpression (**B**,**D**,**F**) in indicated cell lines. (**G**–**L**) Apoptosis rate measured by flow cytometry after ITGA2 knockdown (**G**,**I**,**K**) or overexpression (**H**,**J**,**L**). The total apoptosis rate (sum of early and late apoptosis) is delineated by the yellow boxes. (**M**–**R**) Cell cycle distribution analyzed by flow cytometry after ITGA2 knockdown (**M**,**O**,**Q**) or overexpression (**N**,**P**,**R**). (**A**–**R**) All the cells were treated with an appropriate concentration of lenvatinib (knockdown groups: Hep3B-LR, Huh7-LR, and SNU449 with lenvatinib at a concentration of 10 μM; overexpression groups: Hep3B and Huh7 with lenvatinib at a concentration of 1 μM; SNU449 with lenvatinib at a concentration of 15 μM). * *p* < 0.05, ** *p* < 0.01, *** *p* < 0.001, **** *p* < 0.0001.

Given that ITGA2 affects cell proliferation, we further investigated whether ITGA2 influences cell growth by regulating apoptosis and the cell cycle. Western blotting for apoptosis-related proteins revealed that after ITGA2 knockdown in drug-resistant cells (Hep3B-LR, Huh7-LR, and SNU449) and their treatment with lenvatinib, the expression of the proapoptotic proteins Bax and Caspase-3 was upregulated, whereas the expression of the antiapoptotic protein Bcl-2 was downregulated (Figure 3A,C,E). Flow cytometry analysis further confirmed that knocking down ITGA2 significantly increased the rate of lenvatinib-induced apoptosis (Figure 3G,I,K). Conversely, Western blotting indicated that overexpression of ITGA2 in sensitive cells (Hep3B, Huh7) and SNU449 cells reduced the expression of the proapoptotic proteins Bax and Caspase-3 and increased the expression of the antiapoptotic protein Bcl-2 (Figure 3B,D,F), and flow cytometry further revealed a significant reduction in the rate of apoptosis induced by lenvatinib (Figure 3H,J,L). The results of the Western blotting and flow cytometry experiments consistently demonstrated that the expression of ITGA2 was negatively correlated with lenvatinib-induced apoptosis. In terms of the cell cycle, flow cytometry analysis revealed that, compared with control cells, drug-resistant cells in which ITGA2 was knocked down exhibited more significant G1 phase arrest (Figure 3M,O,Q). When ITGA2 was overexpressed in sensitive cells and SNU449 cells, a reduced degree of G1 phase arrest was observed (Figure 3N,P,R). These cell experiments demonstrated that ITGA2 can increase the drug resistance of cells to lenvatinib by reducing lenvatinib-induced apoptosis and cell cycle arrest.

**Figure 4:**
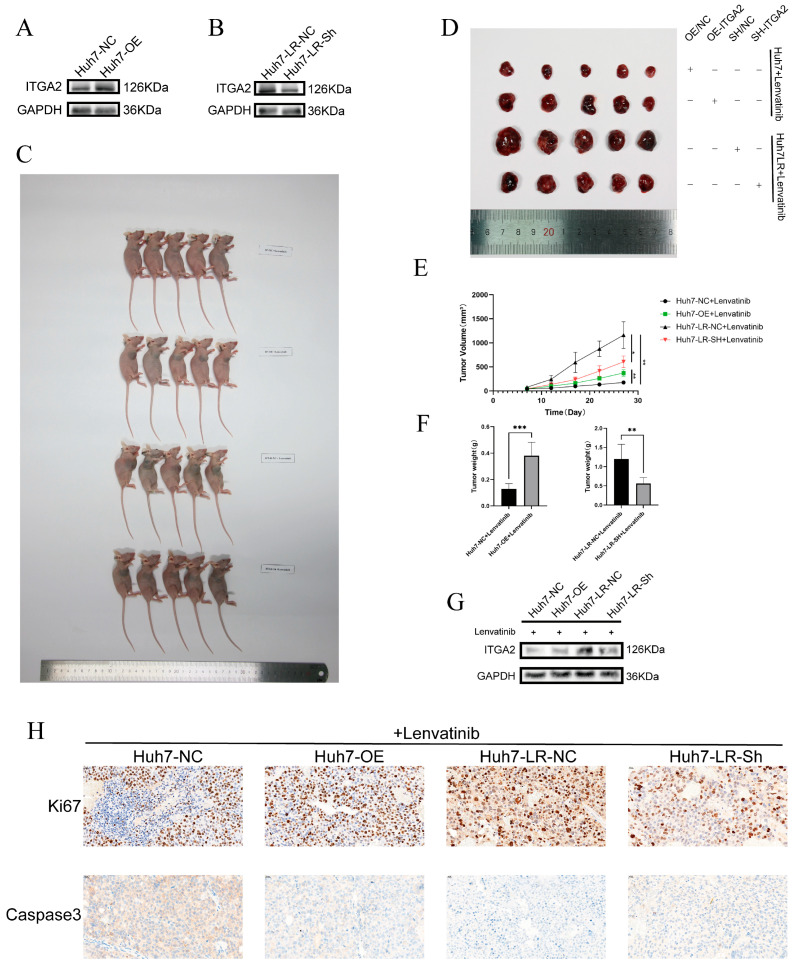
Animal experiments confirmed that ITGA2 increases the resistance of hepatocellular carcinoma to the drug lenvatinib. (**A**,**B**) WB confirmed the successful construction of stable knockdown Huh7-LR cell lines and stable overexpression Huh7 cell lines for ITGA2. (**C**) Subcutaneous tumor formation experiments in mice were conducted between the Huh7-overexpressing and control groups as well as between the Huh7-LR-knockdown and control groups [*n* = 5]. (**D**) Images of subcutaneous tumors obtained from mice after treatment with lenvatinib (10 mg/kg). (**E**) Tumor growth curves of the Huh7-overexpressing group, Huh7-LR-knockdown group, and corresponding control groups. (**F**) Measurement of tumor weights in the Huh7-overexpressing group, Huh7-LR-knockdown group, and corresponding control groups. (**G**) WB was used to verify the expression level of ITGA2 in tumor tissues. (**H**) Immunohistochemical detection of the expression levels of Ki67 and Caspase-3 in the tumor tissues of each group. * *p* < 0.05, ** *p* < 0.01, *** *p* < 0.001.

To further validate the function of ITGA2 in an in vivo model, we first constructed cells with stable overexpression or knockdown of ITGA2 for subsequent animal experiments. Western blotting confirmed that we successfully generated stable ITGA2-overexpressing Huh7 cells (Huh7-OE) and stable ITGA2-knockdown Huh7-LR cells (Huh7-LR-Sh) and established corresponding control groups with empty vectors (Huh7-NC and Huh7-LR-NC) (Figure 4A,B). These cells were subcutaneously inoculated into BALB/c nude mice, which were subsequently treated with lenvatinib (10 mg/kg). Tumor growth was dynamically monitored, and the subcutaneous tumors were removed and photographed after a certain period (Figure 4C,D). The results were consistent with the trends observed in the cell experiments. The tumor growth rate and size in the Huh7-OE group were significantly greater than those in the Huh7-NC control group. Compared with those in the Huh7-LR-NC control group, the tumor growth rate and size in the Huh7-LR-Sh group were significantly lower (Figure 4E,F). Western blotting further confirmed that we had successfully transplanted stable ITGA2-overexpressing or -knockdown cells (Figure 4G). Immunohistochemical analysis revealed that compared with that in the Huh7-NC control group, the positive rate of the proliferation marker Ki67 in the Huh7-OE group was significantly greater, and the positive rate of the apoptosis marker Caspase-3 was significantly lower. Compared with the Huh7-LR-NC control group, the Huh7-LR-Sh group showed a trend toward a significantly decreased positive rate of Ki67 and a significantly increased positive rate of Caspase-3 (Figure 4H). On the basis of in vitro and in vivo experiments, we confirmed that ITGA2 can lead to a decrease in the drug sensitivity of hepatocellular carcinoma to lenvatinib.

### 3.3. The ITGA2 Inhibitor E7820 Enhances the Sensitivity of Hepatocellular Carcinoma to Lenvatinib

**Figure 5:**
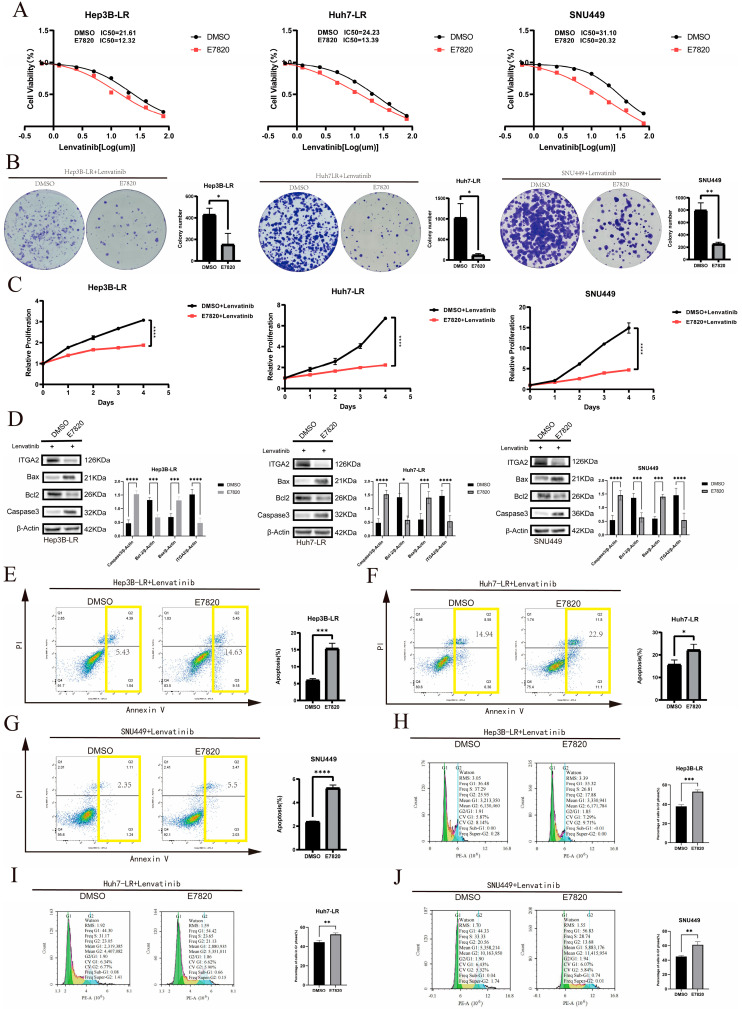
E7820 enhances lenvatinib sensitivity in hepatocellular carcinoma cells. (**A**) CCK8 assays showing reduced IC50 of lenvatinib after E7820 treatment in Hep3B-LR, Huh7-LR, and SNU449 cells. (**B**) Colony formation assays indicating enhanced inhibitory effect of lenvatinib by E7820. (**C**) CCK8 assays demonstrating E7820-promoted suppression of cell proliferation by lenvatinib. (**D**) WB analysis of Bax, Bcl-2, and Caspase-3 expression after combination treatment. (**E**–**G**) Flow cytometry analysis of apoptosis after E7820 and lenvatinib treatment. The total apoptosis rate (sum of early and late apoptosis) is delineated by the yellow boxes. (**H**–**J**) Cell cycle distribution detected by flow cytometry following combination treatment. (**B**–**J**) All the experiments were performed with a lenvatinib concentration of 10 μM. * *p* < 0.05, ** *p* < 0.01, *** *p* < 0.001, **** *p* < 0.0001.

To facilitate future clinical translation studies, we obtained the small molecule inhibitor E7820 for ITGA2. Through in vitro and in vivo experiments, we investigated whether E7820 could increase the sensitivity of HCC to lenvatinib. In the cell experiments, the IC50 value of E7820 in resistant cells (Hep3B-LR, Huh7-LR, and SNU449) was determined, and the synergistic index (CI) of its combination with lenvatinib was calculated. The results revealed that E7820 had a synergistic effect with lenvatinib (CI < 1), and Western blotting confirmed that E7820 effectively reduce the expression of ITGA2 in resistant cells (Supplementary Figure S2). Further CCK8 experiments demonstrated that treatment with E7820 significantly reduced the IC50 value of lenvatinib in resistant cells (Figure 5A), indicating that E7820 enhanced the sensitivity of cells to lenvatinib. Compared with lenvatinib alone, treatment with E7820 combined with lenvatinib significantly inhibited the colony formation ability and viability of resistant cells (Figure 5B,C). Western blotting was used to detect changes in the levels of apoptotic proteins. Compared with those in the control group, the expression of the proapoptotic proteins Bax and Caspase-3 was upregulated, and the expression of the antiapoptotic protein Bcl-2 was downregulated in the E7820 combined with lenvatinib treatment group (Figure 5D). Compared with the control, lenvatinib combined with E7820 significantly increased the rate of apoptosis induced by lenvatinib (Figure 5E–G) and enhanced G1 phase cell cycle arrest (Figure 5H–J).

**Figure 6:**
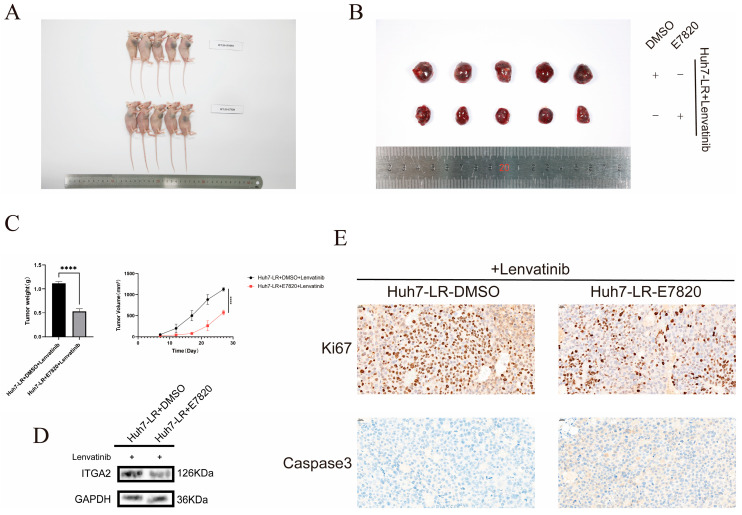
Animal experiments confirmed that E7820 can increase the drug sensitivity of the body to lenvatinib. (**A**) Mouse subcutaneous tumor formation experiments were conducted in the control group and the inhibitor group [*n* = 5]. (**B**) Representative images of subcutaneous tumors in the control group and the inhibitor group after treatment with lenvatinib (10 mg/kg). (**C**) Measurement of tumor growth curves and weights in the control group and the inhibitor group. (**D**) WB verification of the expression level of ITGA2 in tumor tissues. (**E**) Immunohistochemical detection of the expression levels of Ki67 and Caspase-3 in tumor tissues from the control group and the inhibitor group. **** *p* < 0.0001.

A subcutaneous xenograft model was developed in BALB/c nude mice by inoculating them with Huh7-LR cells for the in vivo study. The efficacy of the combination therapy, consisting of lenvatinib (10 mg/kg) and E7820 (5 mg/kg), was subsequently evaluated. The control group was given lenvatinib (10 mg/kg) combined with solvent (DMSO). Compared with those in the lenvatinib monotherapy group, the tumor growth rates of the mice in the lenvatinib combined with E7820 treatment group were significantly lower, and the tumor weights were significantly lower (Figure 6A–C). The Western blotting results indicated that the expression level of ITGA2 in the tumor tissue of the combined treatment group was lower than that in the control group (Figure 6D). Immunohistochemical analysis revealed that the positive rate of Ki67 in the tumor tissue of the combined treatment group was significantly lower, whereas the positive rate of Caspase-3 was significantly higher (Figure 6E). Cell and animal experiments demonstrated that E7820 has a sensitizing effect on hepatocellular carcinoma cells during lenvatinib treatment, providing new ideas and a theoretical basis for the clinical translation of lenvatinib drug sensitization.

### 3.4. The ITGA2/AKT/FOXO3A Signaling Axis Promotes Resistance to Lenvatinib in Hepatocellular Carcinoma

**Figure 7:**
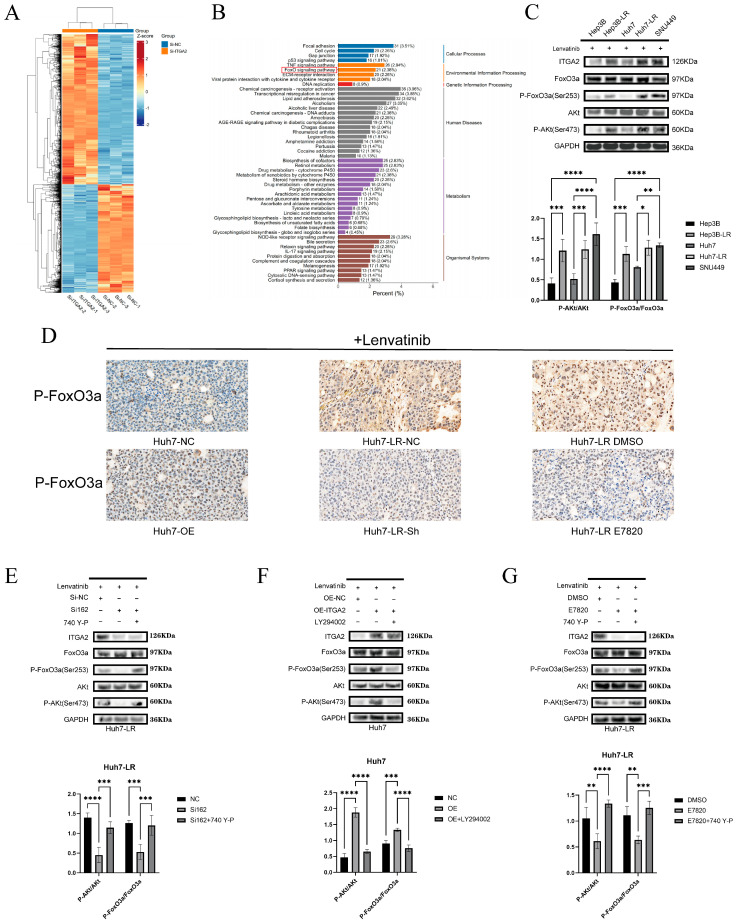
ITGA2 activates the FOXO signaling pathway in hepatocellular carcinoma to promote resistance to lenvatinib. (**A**) Heatmap of differentially expressed genes. (**B**) KEGG enrichment analysis of differentially expressed genes. (**C**) WB analysis of AKT/FOXO pathway-related proteins in Hep3B, Hep3B-LR, Huh7, Huh7-LR, and SNU449 cells treated with lenvatinib (1 μM). (**D**) Immunohistochemical staining of P-FOXO3A in tumor tissues from Huh7-overexpression, Huh7-LR-knockdown, Huh7-LR-inhibitor, and corresponding control groups. (**E**) WB detection of changes in AKT/FOXO pathway-related proteins in the Huh7-LR-knockdown group (control group, knockdown group, and knockdown group treated with 740-YP) (lenvatinib concentration of 10 μM). (**F**) WB detection of changes in AKT/FOXO pathway-related proteins in the Huh7 overexpression group (control group, overexpression group, and overexpression group treated with LY294002) (lenvatinib concentration of 1 μM). (**G**) WB detection of changes in AKT/FOXO pathway-related proteins in the Huh7-LR inhibitor group (control group, inhibitor group, and inhibitor group treated with 740-YP) (lenvatinib concentration of 10 μM). (**A**–**G**) All the experimental groups were treated with the appropriate concentrations of lenvatinib. * *p* < 0.05, ** *p* < 0.01, *** *p* < 0.001, **** *p* < 0.0001.

To further explore the downstream mechanism of ITGA2-mediated drug resistance, we conducted transcriptome sequencing and differential gene analysis on Huh7-LR cells in the ITGA2-knockdown (si*ITGA2*) and control (siNC) groups. The cluster heatmap illustrates the differential expression patterns of the differentially expressed genes (Figure 7A). KEGG pathway enrichment analysis revealed that ITGA2 knockdown significantly affected multiple pathways, including cell adhesion, the cell cycle, the TNF signaling pathway, the p53 signaling pathway, and the FOXO signaling pathway (Figure 7B). Our transcriptome sequencing results suggested that the target genes of *FOXO* in the FOXO signaling pathway were significantly differentially expressed, but the expression of *FOXO* genes did not change significantly (Supplementary Figure S3). The literature suggests that the FOXO pathway is usually regulated by phosphorylated AKT, whose phosphorylation level affects its transcriptional activity. Therefore, our results suggest that ITGA2 activates the AKT/FOXO pathway by regulating its phosphorylation level and mediates lenvatinib resistance in hepatocellular carcinoma. To further verify our speculation, we focused on the activity state of the FOXO pathway. Western blot analysis revealed that the phosphorylation level of FOXO3A in the lenvatinib-resistant cell lines was significantly greater than that in the lenvatinib-sensitive cell lines (Figure 7C). Moreover, we conducted immunohistochemical analysis of previous animal experiments and reported that the positive rate of P-FOXO3A in the Huh7-OE group was higher than that in the corresponding group, whereas the positive rates of P-FOXO3A in the Huh7-LR-Sh group and Huh7-LR-E7820 group were lower than those in the corresponding control groups (Figure 7D). On the basis of the above results, we speculate that ITGA2 activates the AKT signaling pathway to promote the phosphorylation of FOXO3A, leading to the inactivation of FOXO3A transcription and the mediation of lenvatinib resistance.

**Figure 8:**
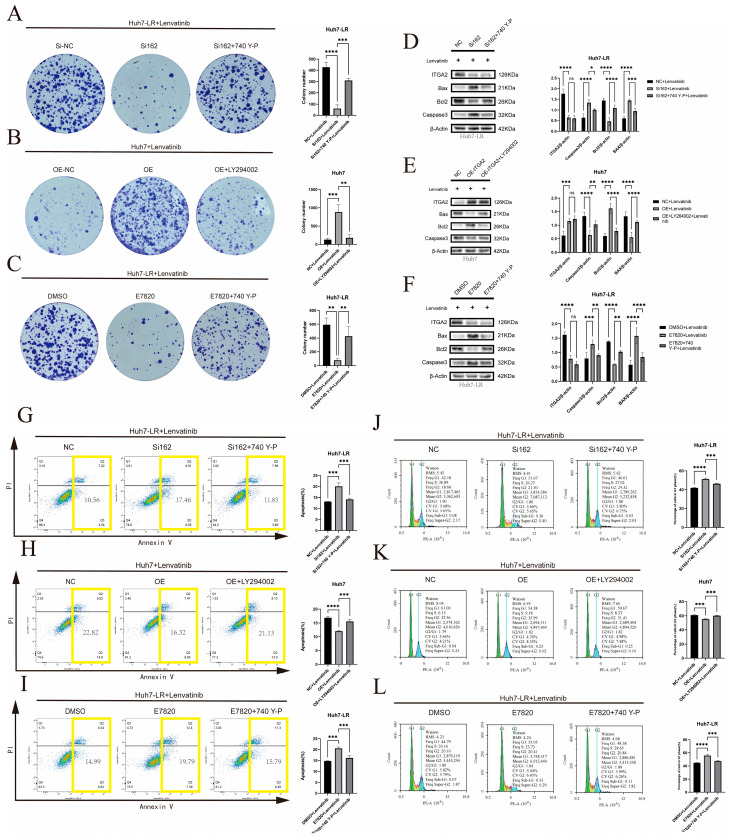
A rescue experiment confirmed that ITGA2 promotes resistance to lenvatinib in hepatocellular carcinoma by activating the FOXO signaling pathway. (**A**–**C**) After treatment with LY294002, Huh7 (**B**) cells with ITGA2 overexpression, Huh7-LR (**A**) cells with 740-YP knockdown, and Huh7-LR (**C**) cells treated with inhibitors were used for colony formation experiments to evaluate the proliferation ability of cells treated with lenvatinib. (**D**–**F**) After treatment with LY294002, ITGA2-overexpressing Huh7 (**E**) cells, 740-YP-treated ITGA2-knockdown Huh7-LR cells (**D**), and Huh7-LR cells treated with inhibitors (**F**) were subjected to WB experiments to detect the expression levels of ITGA2, Bax, Bcl-2, and Caspase-3 in the cells treated with lenvatinib. (**G**–**I**) After treatment with LY294002, Huh7 (**H**) cells with ITGA2 overexpression, 740-YP-treated ITGA2-knockdown Huh7-LR cells (**G**), and Huh7-LR (**I**) cells treated with the inhibitor were subjected to flow cytometry to detect the rate of apoptosis in response to lenvatinib treatment. The total apoptosis rate (sum of early and late apoptosis) is delineated by the yellow boxes. (**J**–**L**) After treatment with LY294002, ITGA2-overexpressing Huh7 (**K**) cells, 740-YP-treated ITGA2-knockdown Huh7-LR (**J**) cells, and Huh7-LR (**L**) cells treated with the inhibitor were subjected to flow cytometry to detect the cell cycle during lenvatinib treatment. (**A**–**L**) All the cells were treated with the appropriate concentrations of lenvatinib (1 μM lenvatinib for the overexpression group, 10 μM lenvatinib for the knockdown group, and 10 μM lenvatinib for the inhibitor group). * *p* < 0.05, ** *p* < 0.01, *** *p* < 0.001, **** *p* < 0.0001. ns, not significant.

To clarify the necessity of the AKT/FOXO3A pathway in ITGA2-mediated resistance, we conducted functional rescue experiments using pathway regulators. Western blotting was used to detect the protein expression levels in each group of cells. The results suggested that after knockdown of ITGA2 in Huh7-LR cells, the phosphorylation levels of AKT (Ser473) and FOXO3A (Ser253) significantly decreased. Administration of the AKT activator 740Y-P restored their phosphorylation levels (Figure 7E). Conversely, overexpression of ITGA2 in Huh7 cells increased the phosphorylation of AKT (Ser473) and FOXO3A (Ser253), and administration of the PI3K/AKT inhibitor LY294002 reversed this effect (Figure 7F). Similarly, treatment of Huh7-LR cells with the E7820 inhibitor also significantly reduced the phosphorylation levels of AKT (Ser473) and FOXO3A (Ser253), and this inhibitory effect was reversed by 740Y-P (Figure 7G). Colony formation experiments revealed that in Huh7-LR cells with ITGA2 knockdown or E7820 treatment, the administration of 740Y-P reversed the proliferative inhibitory phenotype of these cells (Figure 8A,C), whereas in Huh7 cells overexpressing ITGA2, LY294002 eliminated their ability to increase proliferation (Figure 8B). Western blotting for apoptotic proteins revealed that 740Y-P treatment reversed the upregulation of proapoptotic proteins (Bax and Caspase-3) and the downregulation of antiapoptotic proteins (Bcl-2) caused by ITGA2 knockdown or treatment with E7820 (Figure 8D,F), whereas LY294002 treatment reversed the downregulation of proapoptotic proteins and the upregulation of antiapoptotic proteins caused by ITGA2 overexpression (Figure 8E). The flow cytometry results consistently revealed that 740Y-P treatment significantly reduced the increase in apoptosis and G1 phase arrest induced by ITGA2 knockdown or treatment with E7820 (Figure 8G,I), whereas LY294002 treatment reversed the reduction in apoptosis and weakening of G1 phase arrest caused by ITGA2 overexpression (Figure 8H). These rescue experiments demonstrated that ITGA2 mainly promotes lenvatinib resistance by activating the AKT/FOXO3A signaling pathway.

## 4. Discussion

Liver cancer is the sixth most common type of cancer worldwide, and hepatocellular carcinoma (HCC) constitutes the majority of primary liver malignancies. Among the targeted therapies for hepatocellular carcinoma, tyrosine kinase inhibitors (TKIs), such as lenvatinib and sorafenib, are the first-line options for advanced patients. Lenvatinib has a comparable overall survival rate to that of sorafenib and has become the more common choice [5,6]. However, its efficacy is often limited by drug resistance. Therefore, it is crucial to study the resistance mechanisms involved and propose effective treatment strategies.

Integrins are heterodimers composed of α and β subunits that primarily function to mediate cell adhesion to extracellular matrices such as collagen and participate in the regulation of cell migration, signal transduction, and microenvironment homeostasis. Moreover, integrins are common factors contributing to cancer drug resistance, including resistance to tyrosine kinase inhibitors (TKIs) [19,20]. To investigate whether integrins are the cause of TKI drug resistance, we conducted bioinformatics prediction of the correlations between each subunit and TKI drug resistance and found that ITGA2 was significantly correlated with lenvatinib resistance. To better understand the molecular mechanism of resistance, on the basis of the conventional construction of lenvatinib-induced acquired resistance models, we also selected primary resistance models [21]. In lenvatinib-resistant cells, high expression of ITGA2 was significantly associated with lenvatinib resistance. Previous studies have reported that ITGA2 is closely related to chemotherapy sensitivity and that its high expression mediates gemcitabine resistance in pancreatic cancer [13], 5-FU resistance in colorectal cancer [22], and 5-FU and ADR resistance in gastric cancer [23]. These findings also indicate that abnormal expression of ITGA2 is closely related to the progression of various malignant tumors. For example, based on bioinformatics analysis, ITGA2 was predicted to be associated with the prognosis of liver cancer [24]; in intrahepatic cholangiocarcinoma, elevated ITGA2 expression promotes type I collagen-induced clonogenic growth of tumor cells [25]; in esophageal squamous cell carcinoma, ITGA2 promotes cell proliferation and epithelial–mesenchymal transition (EMT) through the FAK/AKT pathway, and its high expression can significantly increase the invasion ability of tumor cells [26]. High expression of ITGA2 is associated with poor prognosis in pancreatic cancer patients, and the mechanism involves the upregulation of PD-L1 and remodeling of the immune microenvironment [27]. Additionally, ITGA2 has been confirmed to increase the proliferation and metastatic potential of various tumor cells by activating the STAT3 signaling pathway [28]. However, to date, no studies have explored the role of ITGA2 in the sensitivity of liver cell carcinoma to lenvatinib or the mechanism involved. Compared with the control, knockdown of ITGA2 promoted apoptosis and cell cycle arrest, whereas overexpression of ITGA2 reduced apoptosis and cell cycle arrest, promoting cell growth and confirming that ITGA2 promotes lenvatinib drug resistance in liver cell carcinoma.

To further explore the specific mechanism by which ITGA2 mediates resistance to lenvatinib, we conducted transcriptome sequencing on Huh7-LR cells with ITGA2 knocked down and control cells to detect the genes that were differentially expressed between the two groups and performed pathway enrichment analysis. The results revealed that the FOXO signaling pathway was one of the significantly regulated pathways. By analyzing the KEGG signaling pathway map, we found that there was no significant difference in the expression of *FOXO* genes in the FOXO signaling pathway, but the expression of its downstream target genes changed significantly. On the basis of the literature, we found that FOXO is regulated mainly by AKT and its phosphorylation level, thereby affecting its transcriptional activity [29,30]. Therefore, the expression of the *FOXO* gene may not change significantly. FOXO3A is a key transcription factor in the FOXO pathway, and its activity is regulated by AKT phosphorylation. Previous studies have shown that FOXO3A regulates the proliferation, apoptosis, and cell cycle progression of malignant tumors [31,32,33]. Moreover, it has been reported that FOXO3A can regulate chemotherapy and targeted drug resistance; for example, in cholangiocarcinoma, FOXO3A inactivation through the Keap1–Nrf2 axis leads to chemotherapy resistance [34]. In lung cancer, AKT-induced increased nuclear export of FOXO3A leads to multidrug resistance [35]. In gastric cancer, silencing CALM2 can increase sensitivity to afatinib by regulating the AKT/FOXO3A/Puma axis [36]. However, no studies have suggested that FOXO3A can regulate sensitivity to lenvatinib. Although the expression of the *FOXO* gene does not change significantly, ITGA2 can promote the phosphorylation of FOXO3A by activating the AKT signaling pathway, thereby leading to the inactivation of FOXO3A transcription and the mediation of lenvatinib resistance. This study established the core regulatory role of the ITGA2-AKT-FOXO3A signaling axis in a lenvatinib-resistant model of HCC. The expression and activity of ITGA2, an integrin α2 subunit, are precisely orchestrated by numerous upstream signals. At the transcriptional level, BACH1 can promote lung adenocarcinoma cell metastasis by directly activating ITGA2 transcription [37], and KLF7 has been shown to regulate ITGA2 expression to maintain oral cancer stem cell properties [38]. Epigenetically, YY1 can recruit HDAC1 to form a repressor complex that downregulates HOXD3 expression, thereby suppressing the ITGA2 pathway in hepatocellular carcinoma cells [39]. Meanwhile, the long non-coding RNA AC018926.2 has been demonstrated to regulate periodontal ligament cell differentiation via the ITGA2/FAK/AKT pathway [40]. At the post-translational level, ADAR1 p110 enhances hepatocellular carcinoma cell adhesion to the extracellular matrix by upregulating ITGA2 expression [41]. Furthermore, the HMGA2–FOXL2 axis has been implicated in regulating metastatic processes in drug-resistant gastric cancer, suggesting a potential indirect modulation of ITGA2 function [42]. Beyond upstream regulation, ITGA2 is also extensively involved in other pathways related to drug resistance and malignant progression. Notably, studies have shown that ITGA2 overexpression upregulates PD-L1 expression through activation of the STAT3 signaling pathway in several malignant tumor cells [28], providing direct evidence for ITGA2-mediated remodeling of the immune microenvironment. Additionally, ITGA2 inhibits the activation of the TGF-β pathway via the TFCP2–SMAD2 axis, thereby promoting progression in pancreatic cancer [43]. Together, these findings highlight ITGA2’s central role within the tumor microenvironment and multifactorial signaling network: not only is it finely regulated upstream, but it also extensively engages with key pathways such as PD-L1 and TGF-β, potentially collectively mediating drug resistance. Future studies will focus on elucidating the upstream regulatory mechanisms of ITGA2 in lenvatinib resistance in hepatocellular carcinoma and its crosstalk with other resistance-related pathways, thereby systematically clarifying the comprehensive regulatory network of ITGA2 in lenvatinib resistance.

Compared with single-drug therapy, combination therapy is very common, and in many cases has a lower risk of drug resistance [44]. We explored the effect of the ITGA2 small molecule inhibitor E7820 on the drug sensitivity of liver cell carcinoma to lenvatinib to facilitate further clinical translation. In vitro experiments revealed that E7820 treatment significantly reduced the half-maximal inhibitory concentration (IC50) of lenvatinib in resistant liver cancer cells and enhanced the apoptosis and G1 phase arrest of liver cancer cells caused by lenvatinib. In the in vivo model, the combined treatment group presented a greater reduction in tumor volume than the single drug group did, accompanied by a decrease in the Ki67-positive rate and an increase in the Caspase-3-positive rate. This study confirmed that the ITGA2 small molecule inhibitor E7820 has a significant synergistic effect on the antitumor action of lenvatinib. E7820, an oral inhibitor of ITGA2, has completed a phase I clinical trial in patients with advanced solid malignancies, and its pharmacokinetic profile and safety have been preliminarily evaluated [45,46]. Studies demonstrated that E7820 is well absorbed following oral administration, exhibits a moderate half-life compatible with once-daily dosing, and shows dose-proportional increases in both the area under the concentration-time curve (AUC) and the maximum concentration (Cmax) [45,46]. Regarding toxicity, the most common adverse events associated with E7820 monotherapy were mild to moderate fatigue, nausea, and diarrhea, while hematological abnormalities (neutropenia and thrombocytopenia) were the most frequently observed dose-limiting toxicities (DLTs) [45,46]. These findings lay the foundation for future research on combination therapy. E7820 has synergistic effects with other drugs in various cancer models. In non-small cell lung cancer, E7820 combined with erlotinib can synergistically increase the sensitivity of erlotinib-resistant cells to treatment [47]; in osteosarcoma, E7820 combined with cisplatin shows potential therapeutic benefits [48]; and in colorectal cancer models, E7820 combined with chemotherapy drugs can effectively increase the efficacy of chemotherapy [49]. However, there are no reports of combined treatment studies of E7820 and lenvatinib in the field of hepatocellular carcinoma (HCC). This combination provides a new treatment option for patients for whom treatment with lenvatinib alone is insufficient in clinical practice.

This study confirmed that high expression of ITGA2 is a key driving factor for resistance to lenvatinib and that targeting ITGA2 can effectively reverse resistance. We further propose that ITGA2 expression levels may serve as a potential biomarker for patient stratification. Specifically, in hepatocellular carcinoma (HCC) patients with high ITGA2 expression, a first-line treatment strategy combining lenvatinib with an ITGA2 inhibitor may potentially prevent or delay the acquisition of drug resistance. Dynamic monitoring of ITGA2 expression levels also has clinical value in guiding treatment. If the expression of ITGA2 further increases in patients with resistance, switching to a combined treatment regimen at this time may have greater therapeutic benefits. Although this study has confirmed that ITGA2 mediates lenvatinib resistance in hepatocellular carcinoma via the AKT/FOXO3A signaling pathway, several limitations remain, and future studies should focus on the following aspects for further expansion. First, the precise regulatory mechanism between ITGA2 and this signaling pathway has not been fully elucidated. For instance, whether ITGA2 directly regulates AKT phosphorylation or FOXO3A nucleocytoplasmic shuttling requires validation through more precise molecular experiments, such as co-immunoprecipitation (Co-IP), chromatin immunoprecipitation (ChIP), or fluorescent reporter assays. Second, ITGA2 may crosstalk with other known resistance mechanisms, such as drug efflux pumps, DNA repair activation, or tumor microenvironment adaptation, which warrants systematic exploration of its synergistic resistance network via multi-omics integration or functional screening approaches.

## 5. Conclusions

This study systematically revealed the molecular mechanism by which ITGA2 mediates resistance to lenvatinib in HCC through activating the AKT/FOXO3A signaling pathway. Moreover, the application of the ITGA2 small-molecule inhibitor E7820 confirmed that targeting ITGA2 can effectively reverse resistance. This discovery not only deepens the understanding of the mechanism of the integrin family in TKI resistance but also provides new biomarkers and therapeutic targets for lenvatinib resistance in HCC. Furthermore, for subsequent clinical translation, the individualized combined treatment strategy based on the ITGA2 expression level of E7820 and lenvatinib that we describe is expected to constitute a new breakthrough for improving the treatment of advanced HCC.

## Data Availability

The data generated in this study are available upon request from the corresponding author.

## Supplementary Materials

The following supporting information can be downloaded at: https://www.mdpi.com/article/10.3390/cancers17172846/s1.

## Abbreviations

The following abbreviations are used in this manuscript:

HCC	Hepatocellular Carcinoma
ICC	Intrahepatic Cholangiocarcinoma
cHCC-CCA	Combined Hepatocellular-Cholangiocarcinoma
TACE	Transarterial Chemoembolization
TKI	Tyrosine Kinase Inhibitor
ECM	Extracellular Matrix
GSCA	Gene Set Cancer Analysis
CTRP	The Cancer Therapeutics Response Portal
WB	Western Blotting
CCK-8	Cell Counting Kit-8
PVDF	Polyvinylidene Fluoride
FBS	Fetal Bovine Serum
SPF	Specific Pathogen-Free
IACUC	Institutional Animal Care and Use Committee
OS	Overall Survival
PFS	Progression-Free Survival
IC50	Half-Maximal Inhibitory Concentration
CI	Combination Index
EMT	Epithelial-Mesenchymal Transition
FAK	Focal Adhesion Kinase
KEGG	Kyoto Encyclopedia of Genes and Genomes
siRNA	Small Interfering RNA
shRNA	Short Hairpin RNA
KO	Knockout
