# Supplementary material for: ITGA2 Mediates the Resistance of Hepatocellular Carcinoma to Lenvatinib by Activating the AKT/FOXO3A Signaling Pathway
Source: Cancers (Basel). 2025 Aug 29;17(17):2846. doi: 10.3390/cancers17172846 (PMC12427277; doi:10.3390/cancers17172846)

Supplementary Methods S1

Detailed information on shRNA

	sequence
NC shRNA	5′ -GTTCTCCGAACGTGTCACGT-3′
ITGA2-shRNA1	5′ -GGTGTTAGCGCTCAGTCAA -3′
ITGA2-shRNA2	5′ -GCAGTTCTTGGGTACTTAA -3′

Detailed information on siRNA

	sequence	
	sense（5'-3'）	antisense（5'-3'）
ITGA2-Homo-162	GGUGUUAGCGCUCAGUCAATT	UUGACUGAGCGCUAACACCTT
ITGA2-Homo-877	GGAGCAAUUCAAUAUGCAATT	UUGCAUAUUGAAUUGCUCCTT
ITGA2-Homo-927	GCGACGAAGUGCUACGAAATT	UUUCGUAGCACUUCGUCGCTT
ITGA2-Homo-1045	GCAGUUCUUGGGUACUUAATT	UUAAGUACCCAAGAACUGCTT

Detailed information on Overexpression plasmid

	sequence（5'-3'）	bp
ITGA2-Homo	"ITGA2 integrin subunit alpha 2 [Homo sapiens (human)] Gene ID: 3673, updated on 9-Feb-2025 Also known as BR; GPIa; CD49B; HPA-5; VLA-2; VLAA2 Homo sapiens integrin subunit alpha 2 (ITGA2), transcript variant 1, mRNA NCBI Reference Sequence: NM_002203.4"	3546

Detailed information on Overexpression lentivirus

GCCGTTTTTGGCTTTTTTGTTAGACGAAGCTTGGGCTGCAGGTCGACTCTAGAGGATCC
CGCCACCATGGGGCCAGAACGGACAGGGGCCGCGCCGCTGCCGCTGCTGCTGGTGTT
AGCGCTCAGTCAAGGCATTTTAAATTGTTGTTTGGCCTACAATGTTGGTCTCCCAGAAG
CAAAAATATTTTCCGGTCCTTCAAGTGAACAGTTTGGCTATGCAGTGCAGCAGTTTATA
AATCCAAAAGGCAACTGGTTACTGGTTGGTTCACCCTGGAGTGGCTTTCCTGAGAACC
GAATGGGAGATGTGTATAAATGTCCTGTTGACCTATCCACTGCCACATGTGAAAAACTA
AATTTGCAAACTTCAACAAGCATTCCAAATGTTACTGAGATGAAAACCAACATGAGCC
TCGGCTTGATCCTCACCAGGAACATGGGAACTGGAGGTTTTCTCACATGTGGTCCTCT
GTGGGCACAGCAATGTGGGAATCAGTATTACACAACGGGTGTGTGTTCTGACATCAGT
CCTGATTTTCAGCTCTCAGCCAGCTTCTCACCTGCAACTCAGCCCTGCCCTTCCCTCAT
AGATGTTGTGGTTGTGTGTGATGAATCAAATAGTATTTATCCTTGGGATGCAGTAAAGA
ATTTTTTGGAAAAATTTGTACAAGGCCTGGATATAGGCCCCACAAAGACACAGGTGGG
GTTAATTCAGTATGCCAATAATCCAAGAGTTGTGTTTAACTTGAACACATATAAAACCA
AAGAAGAAATGATTGTAGCAACATCCCAGACATCCCAATATGGTGGGGACCTCACAAA
CACATTCGGAGCAATTCAATATGCAAGAAAATATGCTTATTCAGCAGCTTCTGGTGGGC
GACGAAGTGCTACGAAAGTAATGGTAGTTGTAACTGACGGTGAATCACATGATGGTTC
AATGTTGAAAGCTGTGATTGATCAATGCAACCATGACAATATACTGAGGTTTGGCATAG
CAGTTCTTGGGTACTTAAACAGAAACGCCCTTGATACTAAAAATTTAATAAAAGAAATA
AAAGCAATCGCTAGTATTCCAACAGAAAGATACTTTTTCAATGTGTCTGATGAAGCAGC
TCTACTAGAAAAGGCTGGGACATTAGGAGAACAAATTTTCAGCATTGAAGGTACTGTT
CAAGGAGGAGACAACTTTCAGATGGAAATGTCACAAGTGGGATTCAGTGCAGATTACT
CTTCTCAAAATGATATTCTGATGCTGGGTGCAGTGGGAGCTTTTGGCTGGAGTGGGACC
ATTGTCCAGAAGACATCTCATGGCCATTTGATCTTTCCTAAACAAGCCTTTGACCAAAT
TCTGCAGGACAGAAATCACAGTTCATATTTAGGTTACTCTGTGGCTGCAATTTCTACTG
GAGAAAGCACTCACTTTGTTGCTGGTGCTCCTCGGGCAAATTATACCGGCCAGATAGT
GCTATATAGTGTGAATGAGAATGGCAATATCACGGTTATTCAGGCTCACCGAGGTGACC
AGATTGGCTCCTATTTTGGTAGTGTGCTGTGTTCAGTTGATGTGGATAAAGACACCATT
ACAGACGTGCTCTTGGTAGGTGCACCAATGTACATGAGTGACCTAAAGAAAGAGGAA
GGAAGAGTCTACCTGTTTACTATCAAAGAGGGCATTTTGGGTCAGCACCAATTTCTTGA
AGGCCCCGAGGGCATTGAAAACACTCGATTTGGTTCAGCAATTGCAGCTCTTTCAGAC
ATCAACATGGATGGCTTTAATGATGTGATTGTTGGTTCACCACTAGAAAATCAGAATTC
TGGAGCTGTATACATTTACAATGGTCATCAGGGCACTATCCGCACAAAGTATTCCCAGA
AAATCTTGGGATCCGATGGAGCCTTTAGGAGCCATCTCCAGTACTTTGGGAGGTCCTTG
GATGGCTATGGAGATTTAAATGGGGATTCCATCACCGATGTGTCTATTGGTGCCTTTGGA
CAAGTGGTTCAACTCTGGTCACAAAGTATTGCTGATGTAGCTATAGAAGCTTCATTCAC
ACCAGAAAAAATCACTTTGGTCAACAAGAATGCTCAGATAATTCTCAAACTCTGCTTC
AGTGCAAAGTTCAGACCTACTAAGCAAAACAATCAAGTGGCCATTGTATATAACATCAC
ACTTGATGCAGATGGATTTTCATCCAGAGTAACCTCCAGGGGGTTATTTAAAGAAAACA

ATGAAAGGTGCCTGCAGAAGAATATGGTAGTAAATCAAGCACAGAGTTGCCCCGAGCA
CATCATTTATATACAGGAGCCCTCTGATGTTGTCAACTCTTTGGATTTGCGTGTGGACAT
CAGTCTGGAAAACCCTGGCACTAGCCCTGCCCTTGAAGCCTATTCTGAGACTGCCAAG
GTCTTCAGTATTCCTTTCCACAAAGACTGTGGTGAGGACGGACTTTGCATTTCTGATCT
AGTCCTAGATGTCCGACAAATACCAGCTGCTCAAGAACAACCCTTTATTGTCAGCAAC
CAAAACAAAAGGTTAACATTTTCAGTAACGCTGAAAAATAAAAGGGAAAGTGCATAC
AACACTGGAATTGTTGTTGATTTTTCAGAAAACTTGTTTTTTGCATCATTCTCCCTGCCG
GTTGATGGGACAGAAGTAACATGCCAGGTGGCTGCATCTCAGAAGTCTGTTGCCTGCG
ATGTAGGCTACCCTGCTTTAAAGAGAGAACAACAGGTGACTTTTACTATTAACTTTGAC
TTCAATCTTCAAAACCTTCAGAATCAGGCGTCTCTCAGTTTCCAAGCCTTAAGTGAAA
GCCAAGAAGAAAACAAGGCTGATAATTTGGTCAACCTCAAAATTCCTCTCCTGTATGAT
GCTGAAATTCACTTAACAAGATCTACCAACATAAATTTTTATGAAATCTCTTCGGATGGG
AATGTTCCTTCAATCGTGCACAGTTTTGAAGATGTTGGTCCAAAATTCATCTTCTCCCT
GAAGGTAACAACAGGAAGTGTTCCAGTAAGCATGGCAACTGTAATCATCCACATCCCT
CAGTATACCAAAGAAAAGAACCCACTGATGTACCTAACTGGGGTGCAAACAGACAAG
GCTGGTGACATCAGTTGTAATGCAGATATCAATCCACTGAAAATAGGACAAACATCTTC
TTCTGTATCTTTCAAAAGTGAAAATTTCAGGCACACCAAAGAATTGAACTGCAGAACT
GCTTCCTGTAGTAATGTTACCTGCTGGTTGAAAGACGTTCACATGAAAGGAGAATACTT
TGTTAATGTGACTACCAGAATTTGGAACGGGACTTTCGCATCATCAACGTTCCAGACAG
TACAGCTAACGGCAGCTGCAGAAATCAACACCTATAACCCTGAGATATATGTGATTGAA
GATAACACTGTTACGATTCCCCTGATGATAATGAAACCTGATGAGAAAGCCGAAGTACC
AACAGGAGTTATAATAGGAAGTATAATTGCTGGAATCCTTTTGCTGTTAGCTCTGGTTGC
AATTTTATGGAAGCTCGGCTTCTTCAAAAGAAAATATGAAAAGATGACCAAAAATCCA
GATGAGATTGATGAGACCACAGAGCTCAGTAGCGGTATGGACTACAAGGATGACGATG
ACAAGGATTACAAAGACGACGATGATAAGGACTATAAGGATGATGACGACAAATGAGC
TAGCACATAACTTACGGTAAATGGCCCGCCTGGCTGACCGCCCAACGACCCCCGCCCA
TTGACGTCAATAGTAACGCCAATAGGGACTTTCCATTGACGTCAATGGGTGGAGTATTT
ACGGTAAACT

Supplementary Methods S2

Detailed information on antibodies and reagents

Reagent	Company	Art.No.	Dilution concentration
ITGA2	Proteintech Group	30703-1-AP	1 ：4000（WB）
Bax	Proteintech Group	60267-1-Ig	1 ：10000（WB）
Bcl-2	Proteintech Group	12789-1-AP	1 ：5000（WB）
Caspase3	PTM BIO	PTM-5752	1 ：1000（WB）
p-FoxO3a（ser253）	CST	13129	1 ：1000（WB）
FoxO3a	CST	12829	1 ：1000（WB）
p-AKt(ser473）	CST	4060	1 ：2000（WB）
AKt	CST	4691	1 ：1000（WB）
β -Actin	Proteintech Group	66009-1-Ig	1 ：20000（WB）
GAPDH	Proteintech Group	10494-1-AP	1 ：20000（WB）
Goat anti-Rabbit IgG antibody	PTM BIO	PTM-6261	1 ：10000（WB）
Goat anti-Mouse IgG antibody	Abbkine	A21010	1 ：25000（WB）
Lenvatinib	MCE	HY-10981	-
E7820	MCE	HY-14571	-
LY294002	MCE	HY-10108	-
740 Y-P	MCE	HY-P0175	-
Caspase3	PTM BIO	PTM-5752	1 ：500(IHC)
Ki67	Servicebio	GB111499-100	1 ：500(IHC)
P-FoxO3A（Ser253）	Affinity	AF3020	1 ：500(IHC)

Supplementary Figure S1

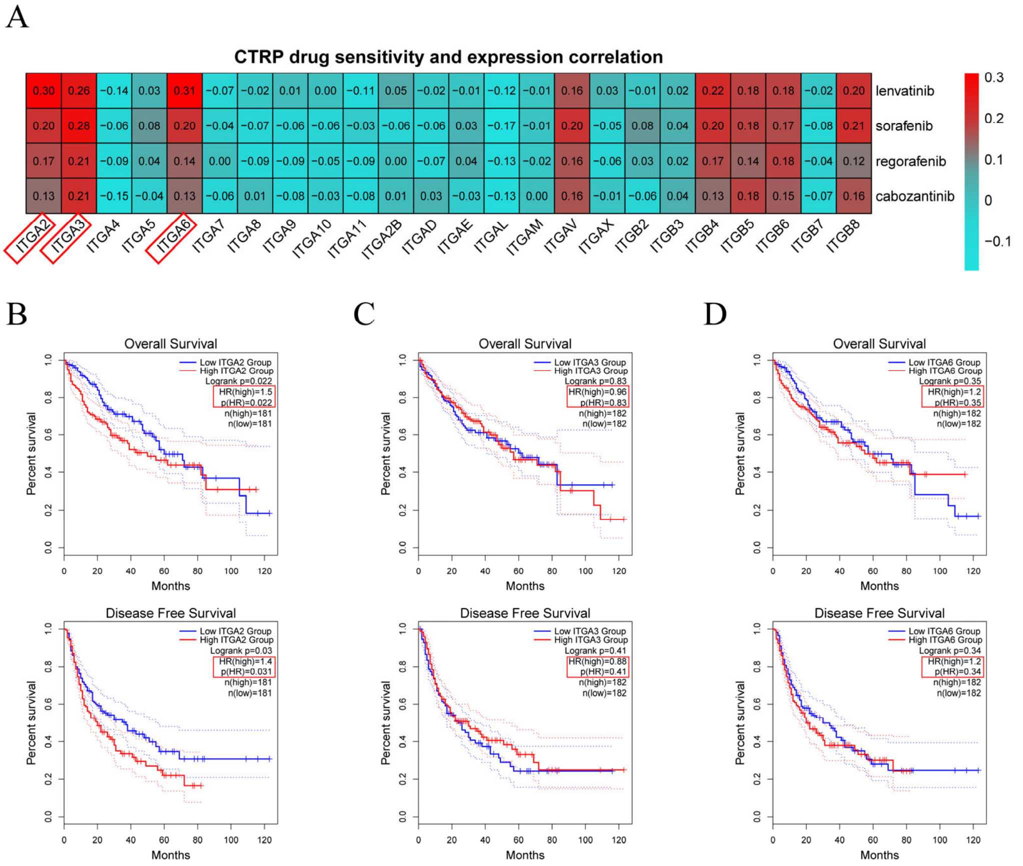

Supplementary Figure S1. ITGA2 has the strongest correlation with resistance to lenvatinib and is most relevant to the prognosis of liver cancer patients.(A) The GSCA website predicts the correlation between different integrin subunits and TKI drug resistance through the CTRP database.(B-D) On the basis of the GEPIA database, the effects of ITGA2 (B), ITGA3 (C), and ITGA6 (D) on survival were predicted, suggesting that the overexpression of ITGA2 is negatively correlated with the prognosis of liver cancer patients.

Supplementary Figure S2

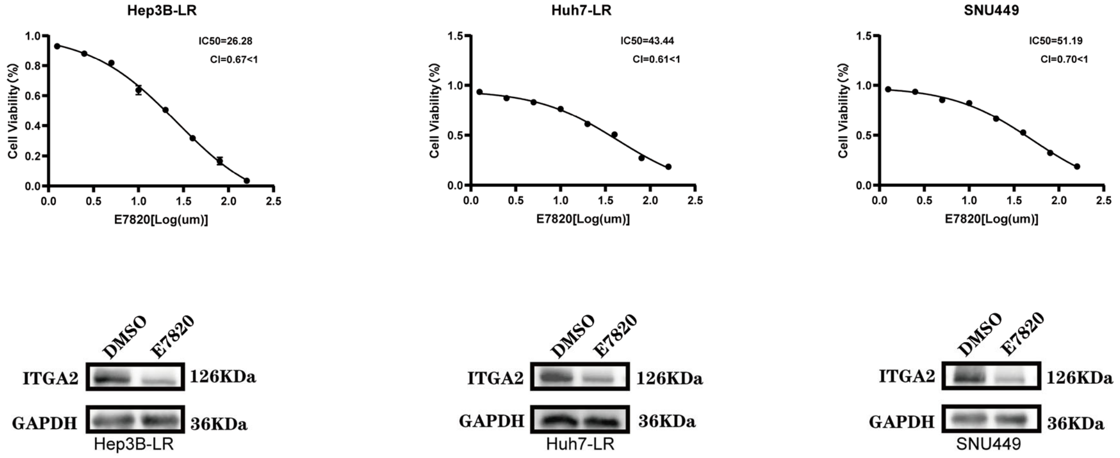

Supplementary Figure 2. A CCK8 assay was used to determine the IC50 of E7820 for lenvatinib in Hep3B-LR,Huh7-LR, and SNU449 cells, and WB was used to verify the decrease in ITGA2 expression.

Supplementary Figure S3

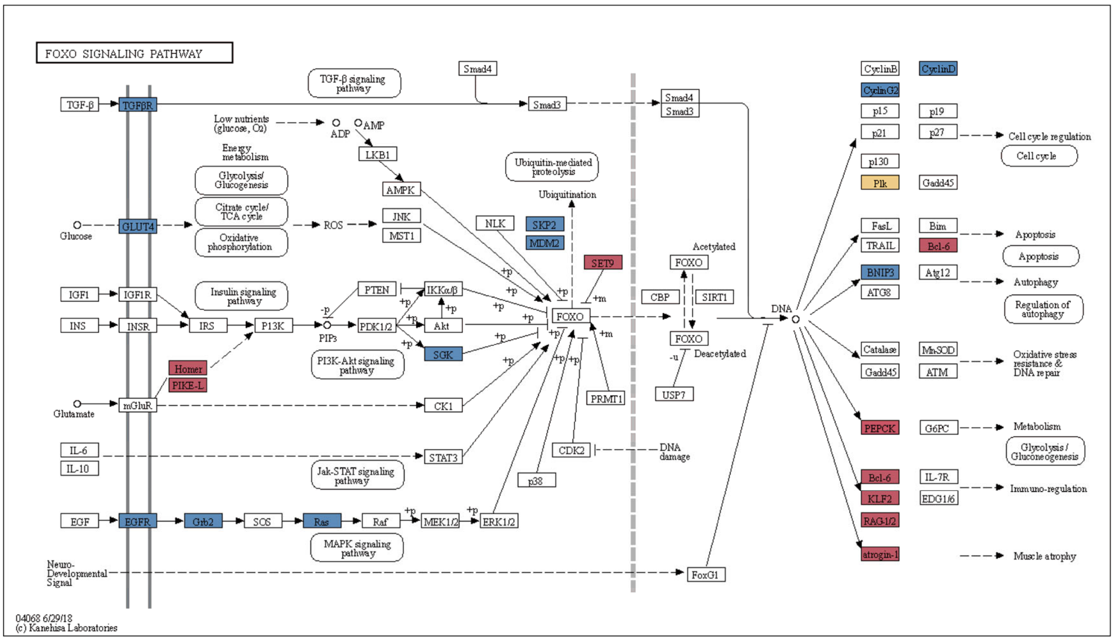

Supplement: Supplementary file 1 [file cancers-17-02846-s001.zip › cancers-3827188-supplementary/Supplementary Materials.pdf]
